# Efficient method for whole‐breast irradiation therapy using Halcyon linear accelerators

**DOI:** 10.1002/acm2.13635

**Published:** 2022-05-19

**Authors:** Yi‐Chun Tsai, Chia‐Chun Wang, Chun‐Wei Wang, Hsiang‐Kung Liang, Shu‐Fan Wang, Chia‐Jung Wu, Chang‐Shiun Lin

**Affiliations:** ^1^ Division of Radiation Oncology, Department of Oncology National Taiwan University Hospital Taipei Taiwan; ^2^ Department of Radiation Oncology National Taiwan University Cancer Center Taipei Taiwan

**Keywords:** breast, efficient, field‐in‐field, Halcyon, hybrid, IMRT

## Abstract

**Background:**

The Halcyon is a linear accelerator‐based treatment machine designed for a high‐throughput simplified workflow. The machine features a compact jawless design, dual‐layer multileaf collimators, and a single 6‐MV flattening filter‐free (FFF) beam. However, the machine's 6‐MV FFF beam may restrict its applicability to conventional techniques, such as field‐in‐field (FiF) radiotherapy, for breast cancer treatment. This study developed a practical and efficient hybrid method for imaging, planning, and irradiation procedures for whole‐breast irradiation using Halcyon linear accelerators.

**Materials and methods:**

The proposed method involves five major steps: (1) field arrangement, (2) planning target volume (PTV) generation and evaluation, (3) basal plan generation, (4) inverse planning intensity–modulated radiation therapy plan generation, and (5) plan evaluation and irradiation. The PTV is generated using isodose curves plotted on the basis of tangential fields, which are applied to create a basal plan. Subsequently, a basal‐dose‐compensation approach is applied to further optimize the treatment plan. This efficient workflow necessitates executing only one onboard cone‐beam computed tomography procedure. This study included 10 patients with early‐stage breast cancer who were treated at our center. The performance of the proposed method was evaluated by comparing its corresponding irradiation time and dose statistics with those derived for a dynamically flattened beam‐based FiF (DFB‐FiF) method.

**Results:**

All plans were normalized to ensure that 98% of the prescribed dose covered 95% of the PTV. On average, the global maximum doses in the proposed and DFB‐FiF methods were lower than 106%. The homogeneity index for right‐sided (left‐sided) breast cancer was 0.053 (0.056) in the proposed method and 0.073 (0.076) in the DFB‐FiF method. The dose statistics of normal tissues, including the contralateral breast, heart, and lungs, were comparable between the methods. However, the irradiation time per monitor unit in the proposed method was approximately five times faster than that in the DFB‐FiF method, but the planning time and complexity were similar between the methods.

**Conclusions:**

This study developed and evaluated an efficient and practical hybrid method for whole‐breast irradiation using the Halcyon. This method can significantly reduce the irradiation time, while providing comparable dose statistics to the DFB‐FiF method.

## 1 INTRODUCTION

Breast cancer is the leading cause of death in women globally.^1^, ^2^ Moreover, breast cancer is the most common cancer in Asian women, and recent data indicate that its incidence in this population is increasing considerably.^3^ In patients with early‐stage breast cancer, radiotherapy is widely used as an adjunct to surgery^4^, ^5^, ^6^ and chemotherapy.

Forward‐planning field‐in‐field (FP FiF) intensity–modulated radiation therapy (IMRT) and inverse planning (IP) IMRT with 6‐ and 10‐MV flattening filter (FF) beams are currently the most common techniques for breast cancer treatment. The FP FiF technique is routinely used at our center because it not only provides a uniform dose distribution in the target region and a short irradiation duration but also obviates the necessity of target delineation. The quality of an FP FiF treatment plan is affected by factors such as subfield weightings, multileaf collimator (MLC) control ability, and breast thickness.^7^ Therefore, the quality and planning time of treatment plans rely on the experience and skills of planners. To improve the planning efficiency and ameliorate the dependence on the skills of planners, IMRT techniques have been introduced for whole‐breast irradiation therapy. By design, such techniques create conformal and homogeneous dose distributions through multiple intensity‐modulated beams.^8^ However, for breast cancer treatment, a pair of opposed tangential fields, namely, medial and lateral fields,^9^, ^10^ are usually applied to spare adjacent normal tissues such as the contralateral breast, heart, and lungs from low‐dose radiation distributions. This scenario may limit the efficacy of the IMRT. In addition, the interplay between the respiratory motion and dynamic MLC sequence introduces additional dose heterogeneity and variations of the target volume coverage. Demonstration of dosimetric effects between planned‐, expected‐dose, and dose per fraction, for shallow, normal, and heavy breathing, was reported. Results indicate that the difference between expected and planned planning target volume (PTV) DVH curves increases with the breathing amplitude.^11^ Moreover, an IMRT field consists of low‐ and high‐monitor unit (MU) segments. In present of organ motion, the low‐MU segments (short delivery time [1–2 s]) can introduce non‐negligible daily dose variation, especially in the penumbra region and for the sliding window technique.^12^ A study proposed a hybrid technique^13^ integrating the use of open and opposed tangential fields (as the first step) and IP IMRT (as the second step) to maintain dose statistics while improving the planning efficiency and robustness for FF beams.

**FIGURE 1:**
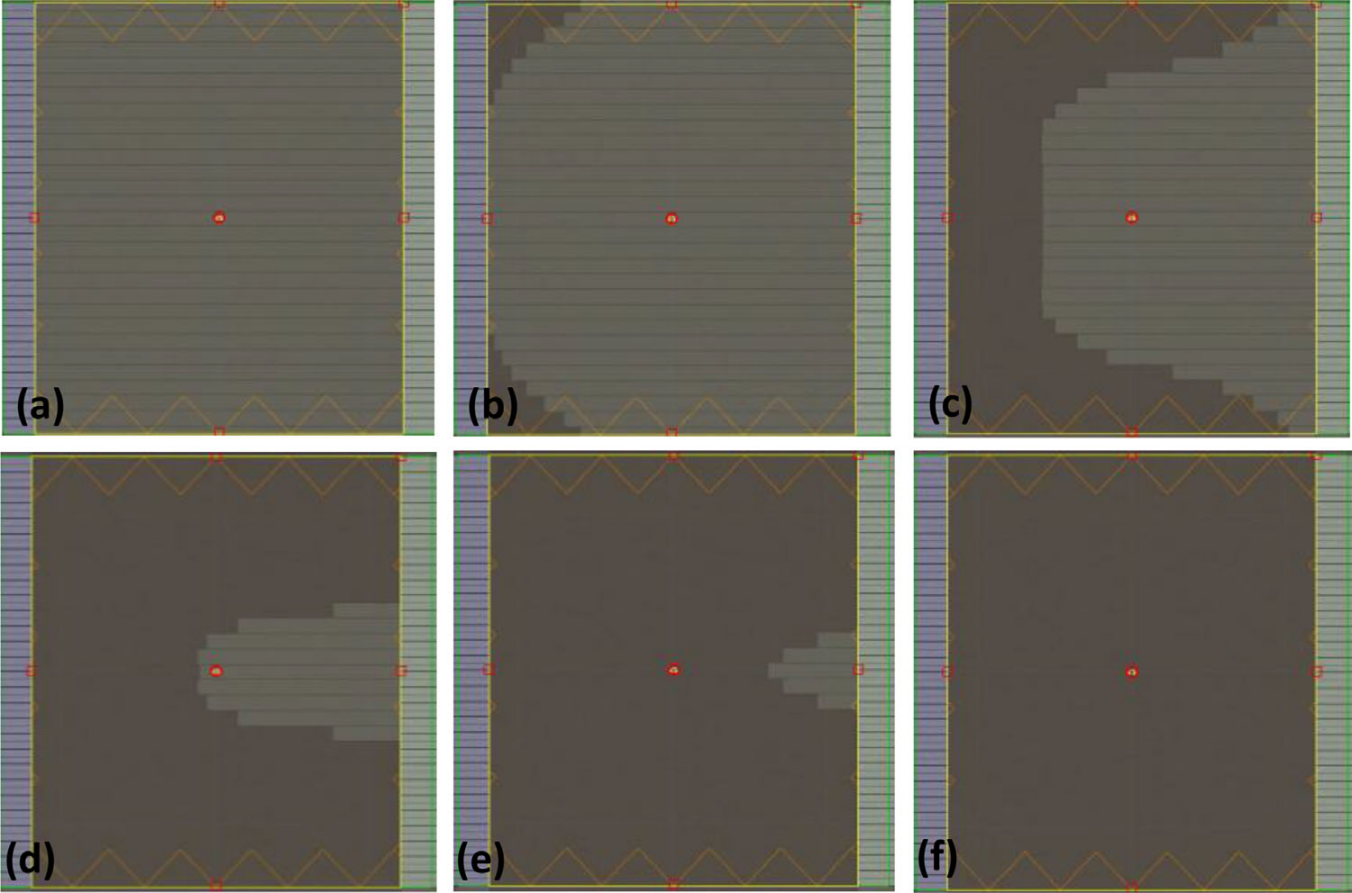
Schematic of DFB MLC sequence generated by the proximal (upper stream) MLC, from (a) to (f), in a 24 cm × 28 cm field. In the field‐in‐field technique, each subfield must have an intact DFB sequence that is inefficient for the irradiation procedure. DFB, dynamically flattened beam; MLC, multileaf collimator

Recently, Varian Medical Systems (Palo Alto, CA, USA) introduced a compact and ring‐shaped linear accelerator (LINAC)‐based machine, namely, Halcyon, to improve clinical efficiency in cancer treatment.^14^, ^15^ Compared with traditional C‐arm LINACs, Halcyon is a jawless machine that does not require field light and is equipped with a double‐layer MLC system. To guarantee positioning accuracy, onboard cone‐beam computed tomography (CBCT) must be executed before irradiation for each plan. In Halcyon, the MLC system is mounted beneath the fixed secondary collimator to provide adequate field shaping and reduce the radiation penetration.^16^ The machine provides 6‐MV FF‐free (FFF) beams with a maximum dose rate of 740 or 800 MUs/min (depending on the calibration condition) at the isocenter. Details regarding the characteristics of the Halcyon machine are provided in the literature.^16^, ^20^ The conventional FiF technique for tangential breast irradiation involves non‐flat beam profiles that inevitably result in the deterioration of dose homogeneity and high‐dose regions in target volumes.^17^ A study proposed a dynamically flattened beam (DFB)‐based method for creating flattened beams in Halcyon.^18^ In this method, flattened profiles are generated by sweeping the proximal MLC (upstream layer) through the entire square field during irradiation, and the distal MLC (downstream layer) executes field shaping. Through this method, the conventional FiF technique can be applied in the Halcyon machine. Morris et al. reported a practical FiF planning technique for tangential breast irradiation (Figure 1).^19^ Compared with 6‐MV FF beams, this technique was reported to have higher MUs and may result in a longer delivery time. Moreover, the technique was reported to exhibit a higher dose rate and faster acceleration and leaf speed, which may compensate for the time cost resulting from non‐flattened beam profiles. However, in the FiF technique for tangential breast irradiation, a flattened beam must be generated for each subfield; that is, the DFB sequence must be repeated for each subfield. This could be the primary explanation for the longer irradiation time in the FiF technique.

This study presents an efficient and practical hybrid method for whole‐breast irradiation using the Halcyon machine. The performance of the proposed method was evaluated by comparing its corresponding delivery time and plan quality—including PTV coverage, global maximum dose, target volume homogeneity, and dose statistics for organs at risk (OARs)—with those of a DFB‐based FiF (DFB‐FiF) method.

## 2 MATERIALS AND METHODS

The Halcyon machine is characterized by a compact size, single 6‐MV FFF beam, fast rotational speed (four rotations per minute), jawless collimator system not requiring field light, double‐layer and staggered MLC field‐shaping technique, and fast leaf speed and acceleration. A Halcyon machine was installed in our center in 2020. The Eclipse (version 16.0; VMS, Palo Alto, CA, USA) treatment planning system is used in our center to create plans. This study developed an efficient hybrid method for both planning and irradiation (including leaf motion). The study also evaluated the performance of the method, including target coverage, homogeneous dose distribution, dose statistics for OARs, and delivery time.

### 2.1 Patient selection

We selected 10 patients with breast cancer who were clinically treated with the DFB‐FiF method or the proposed IP IMRT (hybrid) method from our center. For each patient, two planes, using the DFB‐FiF and hybrid method, were developed. To focus on the planning and delivery efficiency of the methods when used in the Halcyon machine, we included only patients who underwent whole‐breast irradiation. The prescribed PTV dose was either 5000 cGy in 25 fractions (200 cGy/fraction) or 4005 cGy in 15 fractions (267 cGy/fraction). Factors affecting dose statistics, such as breast thickness (distance between the chest wall and skin surface), bridge separation (distance between the medial and lateral borders of the breast tissue), and PTV volume, were recorded and assessed. The prescriptions and geometric characteristics of the included patients are listed in Table 1. Breast size was classified on the basis of PTV volume (343.1–720.3 cm^3^), breast thickness (3.1–6.0 cm), and bridge separation (15.5–20.7 cm).

**TABLE 1:** Prescription and geometric information of patients

Patient ID	Tumor side	PTV vol. (cm^3^)	Separation (cm)	Thickness (cm)	Px. (Gy)	No. Fx
1	Right	424.6	17.4	3.4	40.05	15
2	Right	522.1	17.9	3.6	40.05	15
3	Right	476.0	19.7	3.6	40.05	15
4	Right	720.3	20.4	5.6	40.05	15
5	Left	343.1	15.5	3.1	50.00	25
6	Left	560.0	18.5	4.1	40.05	15
7	Left	389.7	19.0	4.2	40.05	15
8	Left	381.5	19.0	3.2	40.05	15
9	Left	518.3	20.1	3.6	50.00	25
10	Left	640.3	20.7	6.0	50.00	25

Abbreviations: No. Fx., number of fractions; PTV, planning target volume; Px, prescribed dose.

### 2.2 Proposed hybrid method

**FIGURE 2:**
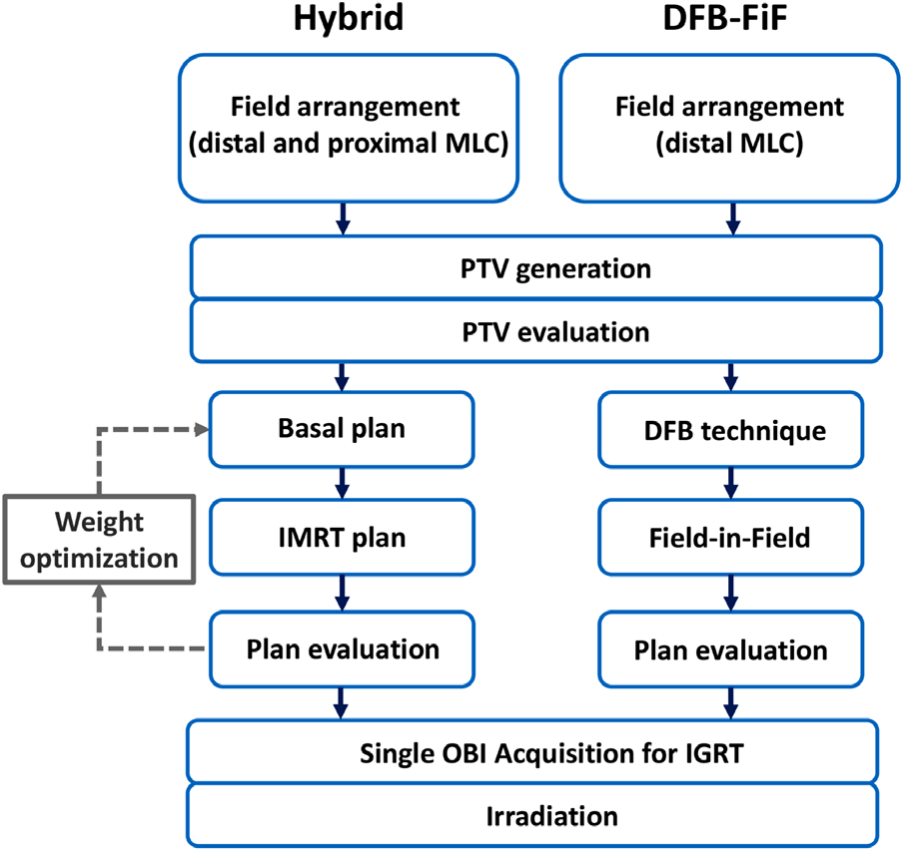
Workflow of the hybrid and DFB‐based FiF methods. The default weight of the basal plan is 70%, and the number of control points of the IMRT plan per field is 166. In this study, weight optimization was not applied because the clinical requirements were met. DFB, dynamically flattened beam; FiF, field‐in‐field; IMRT, intensity‐modulated radiation therapy

The proposed hybrid method involves five major steps (Figure 2): (1) field arrangement, (2) PTV creation, (3) basal plan generation, (4) IP IMRT plan generation, and (5) plan evaluation and irradiation. In the planning procedure, a physician first defines the tangential fields based on beam's‐eye‐view displays (similar to the conventional FiF technique for tangential breast irradiation). A rectangular open field is defined using both the proximal and distal MLCs. In the medial edge, an MLC is used to block the lung region within the irradiated volume to potentially reduce toxicity; moreover, a flash margin of approximately 2 cm is added on the nipple side to provide sufficient fluence in the surrounding air. Subsequently, an opposing field is generated, after which a temporary plan and target volume (PTV_temp_) are generated by applying the prescribed isodose curve (e.g., 50 Gy) to a structure. The physician then evaluates the PTV_temp_ and renames it the “PTV.” In the proposed method, a previously proposed basal‐dose‐compensation (BDC) approach, an optimization technique,^21^ is applied to improve dose homogeneity while maintaining the PTV coverage. A basal plan is generated using equally weighted tangential fields, and the corresponding dose distribution is then used to create a plan for IP IMRT. The field arrangement for IP IMRT was identical to that used for the basal plan. During the BDC progress, the weights between basal and IP IMRT plans are adjusted iteratively. In this study, a weight ranging from 70% to 90% was applied to the basal plan to guarantee the robustness of treatment delivery. In the planning process, plans were optimized using the photon optimizer (PO, version 16.0; VMS, Palo Alto, CA, USA) based on initial dose–volume constraints. The constraints are summarized in Table 2. Moreover, the skin flash tool was applied. Finally, an optimal plan is obtained through the BDC approach for evaluation. In the Halcyon machine, an onboard CBCT system must be executed before treatment to ensure positioning accuracy without the use of a light field. Because the BDC approach creates only a single plan for final evaluation and irradiation, the number of redundant CBCT scanning procedures is significantly reduced.

**TABLE 2:** Summary of initial dose‐volume constraints used in this study

Structure	Limit	Volume (%)	Dose (%)	Priority
Body	Upper	0	102.5	500
PTV	Upper	0	102.5	250
	Lower	98	101.2	200
	Lower	99	99.0	200

Abbreviation: PTV, planning target volume.

### 2.3 DFB‐FiF method

The DFB‐FiF method involves (1) field arrangement, (2) PTV generation, and (3) manual FiF subfield generation. The field arrangement process is similar to that of the hybrid method, except that the field shape is defined using only the distal MLC (downstream). In the Halcyon machine, the proximal MLC generates flattened profiles. Furthermore, the PTV generation process is identical to that of the hybrid method. Additional subfields are created for hot spot reduction with the assistance of digitally reconstructed radiographs. Typically, the MLC placement starts from the lateral edge of each field, and the MLC is moved at steps of approximately 1–2 cm toward the medial direction^17^ for each subfield. To enable the creation of DFB profiles in the proximal MLC, the minimum MU should be restricted to 10 MU per subfield. In general, the number of subfields per field ranges from 3 to 5 to balance the delivery efficiency and dose uniformity.

### 2.4 Dose statistics and delivery efficiency

We compared the plans generated by the hybrid method and DFB‐FiF method in terms of both dose statistics and delivery efficiency. Each plan was normalized to ensure that 98% of the prescribed dose covered 95% of the PTV and to ensure that the global maximum dose (*D*_max_) was less than 107% of the prescribed dose. If *D*_max_ was more than 107%, the normalization was reduced until the value was below 107%. In addition, a homogeneity index (HI) was calculated for PTV by using the following formula: (*D*_2%_–*D*_98%_)/prescribed dose. For OARs, including the contralateral breast, contralateral and ipsilateral lungs, and heart, the maximum and mean doses were analyzed. Moreover, *V*_20Gy_ values derived for the lungs were recorded. To assess the delivery efficiency, including gantry rotation and MLC traveling, the delivery time was measured from the first MU delivered to the end of irradiation. Furthermore, the total MU and number of subfields per plan were recorded.

## 3 RESULTS

Table 3 presents the dose statistics for the PTV and OARs for the proposed hybrid and DFB‐FiF methods. All plans were normalized to meet the clinical requirement; that is, they were normalized to ensure that 98% of the prescribed dose covered 95% of the PTV. The HI derived for the hybrid method was superior to that derived for the DFB‐FiF method. Additionally, the *D*_max_ value derived for the hybrid method was lower than that derived for the DFB‐FiF method by 1.6%. Overall, the dose statistics for OARs were comparable between the two methods. For the heart, the average $D_{2cm^{3}}$ derived for the hybrid method was higher than that derived for the DFB‐FiF method by approximately 5%. The higher $D_{2cm^{3}}$ could be attributed to the IP procedure in the hybrid method.

**TABLE 3:** Summary of dose statistics of PTV and organs at risk

		Right breast				Left breast			
		Hybrid		DFB‐FiF		Hybrid		DFB‐FiF	
Structures	Criteria	Mean	±SD	Mean	±SD	Mean	±SD	Mean	±SD
Body	*D*_max_ (%)	103.8	0.6	105.4	0.9	103.8	0.8	105.4	1.0
PTV	Coverage	98/95		98/95		98/95		98/95	
	HI_mean_	0.056	0.008	0.073	0.008	0.053	0.011	0.076	0.014
Lung, ipsilateral	*D*_max_ (Gy)	39.7	0.5	40.0	0.6	44.5	5.7	44.8	5.6
	*D*_mean_ (Gy)	5.3	0.9	5.3	1.1	6.4	1.1	6.2	1.1
	*V*_20_ (%)	9.5	2.5	8.9	3.1	11.7	2.1	10.7	2.1
Lung, contralateral	*D*_max_ (Gy)	0.3	0.1	0.5	0.1	1.2	0.6	1.3	0.5
	*D*_mean_ (Gy)	0.1	0.0	0.1	0.0	0.1	0.0	0.5	0.9
	*V*_20_ (%)	0.0	0.0	0.0	0.0	0.0	0.0	0.0	0.0
Heart	$D_{2cm^{3}}$ (Px) (%)	5.0	1.4	5.4	1.5	74.0	29.8	69.1	35.2
	*D*_mean_ (Px) (%)	0.9	0.2	1.3	0.3	5.1	3.1	5.4	3.4
Breast, contralateral	*D*_max_ (Gy)	1.4	0.1	1.6	0.1	1.8	0.1	1.9	0.3
	*D*_mean_ (Gy)	0.2	0.0	0.3	0.1	0.2	0.1	0.3	0.1

Abbreviations: $D_{2cm^{3}}$, the minimal dose of 2 cm^3^ of the ORA with the highest dose; DFB‐FiF, dynamically flattened beam–based tangential field‐in‐field method; *D*_max_, global maximum dose; *D*_mean_, averaged dose of the OAR; HI_mean_, HI averaged from all cases; Hybrid, the proposed method; PTV, planning target volume; %Px, percentage of prescribed dose; *V*_20_, volume irradiated exceeding 20 Gy.

**FIGURE 3:**
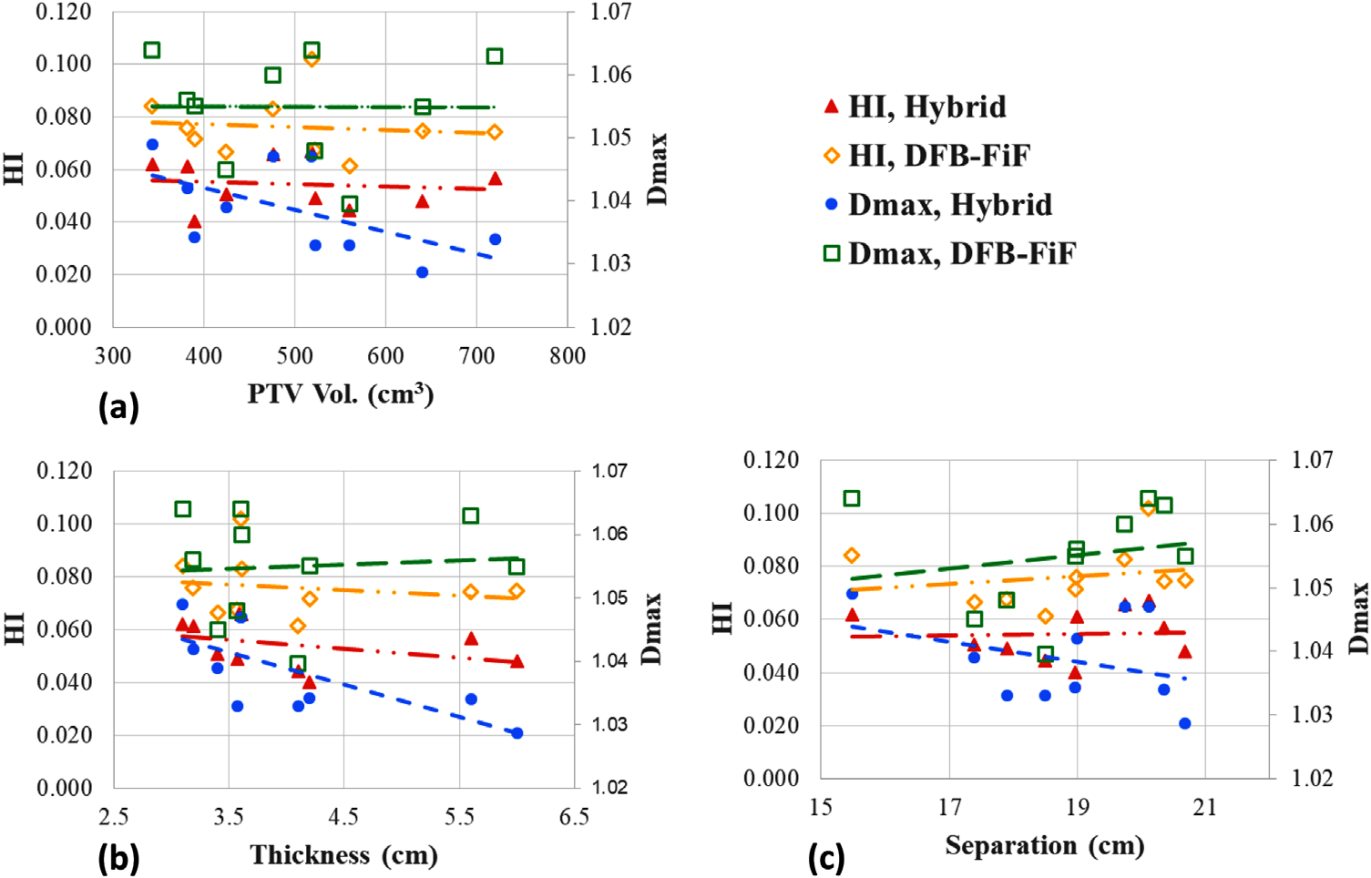
Relationship between PTV (a), breast thickness (b), breast bridge separation (c), HI, and global maximum dose (*D*_max_) of the irradiation volume for the hybrid and DFB‐based FiF methods. The HI of plans of the hybrid and DFB‐FiF methods are represented by red solid triangles (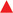) and yellow hollow diamonds (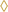), respectively. The *D*_max_ of plans generated by the hybrid and DFB‐FiF methods are represented by blue solid circles (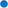) and green hollow squares (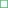), respectively. The trend line generated through the linear fitting of each data set is plotted as well. DFB, dynamically flattened beam; FiF, field‐in‐field; HI, homogeneity index; PTV, planning target volume

The relationship between the HI, *D*_max_, and breast geometry (e.g., PTV, breast thickness, and bridge separation) was investigated for the two methods, and the corresponding data are presented in Figure 3. The prescribed doses were normalized to unity; moreover, a trend line was plotted for each data set. Overall, the hybrid method outperformed the DFB‐FiF method in terms of both the HI (smaller) and *D*_max_ (lower) values for various breast sizes. The results indicated that the HI value derived for the hybrid method was inversely proportional to the PTV and breast thickness and directly proportional to the bridge separation. The *D*_max_ value derived for the DFB‐FiF method increased slightly with an increase in breast size; however, for the hybrid method, the derived *D*_max_ value decreased with an increase in breast size.

**FIGURE 4:**
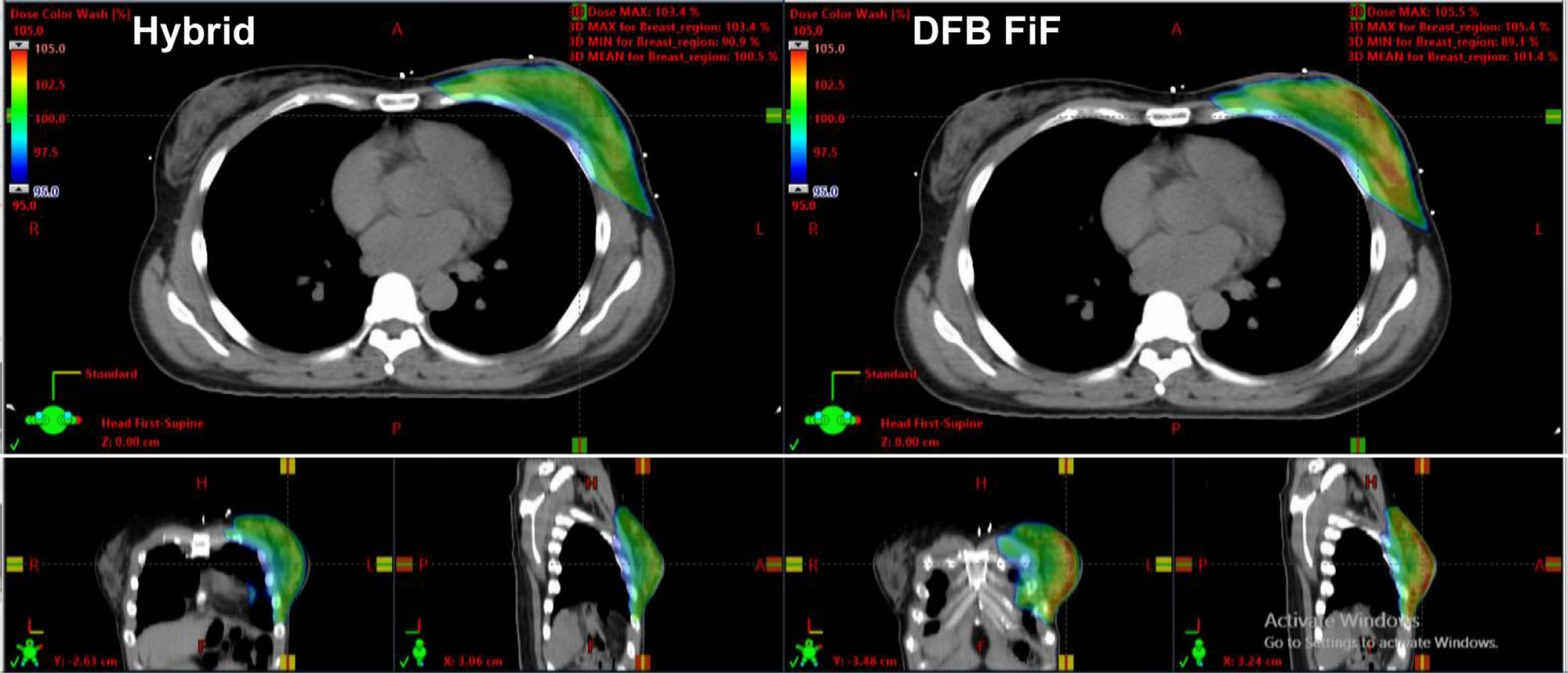
Dose distribution of the generated plans of the hybrid and DFB‐based FiF methods. DFB, dynamically flattened beam; FiF, field‐in‐field

Figure 4 illustrates the dose distributions for the hybrid and DFB‐FiF methods for patient 07 (Table 1). These distributions were determined to be representative of typical plans in terms of the *D*_max_ and coverage values derived for the hybrid and DFB‐FiF methods in the Halcyon machine. The dose uniformity observed for the hybrid method was superior to that observed for the DFB‐FiF method in the axial, sagittal, and coronal views.

Table 4 presents statistics regarding the delivery time, total MU per plan, and the number of subfields (control points) per plan for the two methods. For the DFB‐FiF method, the number of subfields per plan was derived as the summation of the two tangential fields. For the hybrid method, the number of control points for each tangential field was 166; thus, the total number of control points per plan was 332. Compared with the DFB‐FiF method, the hybrid method improved the delivery time per MU by up to 6.5× (range: 3.7×–6.5×); however, the total MU observed for the hybrid method was comparable to that observed for the other technique.

**TABLE 4:** Field information and irradiation time

		Hybrid	DFB‐FiF	Speed‐up/MU^a^
Delivery time (s)	Mean	73	407	4.8
	Min.	65	251	3.7
	Max.	86	524	6.5
Total MU	Mean	595	692	–
	Min.	428	474	–
	Max.	748	841	–
No. of subfield				
Per plan	Mean	332	7	–
	Min.	332	5	–
	Max.	332	8	–

Abbreviations: DFB‐FiF, dynamically flattened beam field‐in‐field; MU, monitor unit.

^a^ Ratio of the delivery time per MU between the DFB‐FiF and hybrid methods.

## 4 DISCUSSION

Overall, the proposed hybrid method outperformed the DFB‐FiF method in terms of HI, but the two methods provided comparable dose statistics. However, for the heart, the average $D_{2cm^{3}}$ value derived for the hybrid method was higher than that derived for the DFB‐FiF method. This could be attributed to the IP IMRT component of the hybrid method, as explained subsequently: A hybrid plan consists of tangents and an IP IMRT plan. During optimization, to achieve desirable coverage, the planning system tends to expand the margin in the medial direction of the PTV. This thus explains why the heart received a higher dose in the hybrid method than it did in the DFB‐FiF method. This could be mitigated by applying additional objectives if necessary. In addition, the hybrid method is designed to improve the dose homogeneity while maintaining the target coverage in present of interplay effect. The dose statistics of the hybrid would be in between the conventional techniques and fully IMRT in general.^13^, ^23^ In this study, the trend of dosimetric data was in agreement with previous works.^13^

In this study, breast size was classified on the basis of the PTV, breast thickness, and bridge separation. The two methods were compared in terms of these classifications, and the results reveal that the *D*_max_ value derived for the hybrid method decreased with an increase in breast size. By contrast, the *D*_max_ value derived for the DFB‐FiF method increased slightly with breast size. Moreover, the HI derived for the hybrid method was smaller than that derived from DFB‐FiF method, as illustrated in Figure 3. These could be attributed to the IP IMRT component of the hybrid method. The number of control points per plan was higher in the hybrid method. By contrast, only four subfields per field were applied in the DFB‐FiF method. According to Figure 3, the breast thickness was more sensitive to HI variation. Additionally, no obvious differences were observed between *D*_max_, and the three breast geometry classifications. Consequently, we suggest that the breast thickness can be applied for similar evaluations.

Halcyon is a compact machine equipped with a single 6‐MV FFF beam, which is characterized by a relatively low mean energy, rapid fall‐off of lateral profiles, and a relatively high dose rate at the isocenter. This machine can perform well for complex IP IMRT applications.^15^, ^22^ However, the non‐flattened profiles of the 6‐MV FFF beam limit the machine's applicability to conventional techniques such as FiF and 2D radiotherapy. A previous study proposed a DFB technique^19^ for generating flattened beams for tangential irradiation in the FiF technique; the study reported that on the basis of the average MU per plan and gantry rotational speed, Halcyon and TrueBeam exhibited similar delivery times. However, the results of the present study reveal that the average delivery time measured for the DFB‐FiF method was 407 s (range: 251–524 s), whereas that measured for the hybrid method was only 73 s (range: 65–86 s). A possible reason for this finding is that the DFB‐FiF method requires a DFB sequence for each subfield. Regarding delivery efficiency, the hybrid method outperformed the DFB‐FiF method while providing comparable planning efficiency. This study developed a hybrid method consisting of two major steps. The first step entails creating a basal plan using a pair of open tangential fields, and the second step involves IP IMRT optimization based on the dose distributions of the basal plan. The weights of the basal plan can be maintained at 70% of the prescribed dose, and the plan can serve as the basis for the development of plans with sufficient dose statistics. The delivery time can be further reduced by increasing the weights of the basal plan, but this would occur at the cost of dose homogeneity. For now, the Eclipse (v16.0.1) supports the number of segments ranging from 64 to 500 (166 by default). Even applying 64 segments to each field of the IP IMRT component, the field is inevitably made up of a numerous amount of very low MU segments (<1 s delivery time). Both robustness and dose statistics of plans could be deteriorated when increasing the weight of IP IMRT component. As a result, it is suggested that the basal plan should take at least 70% of the prescribed dose to allow the proposed method to mitigate the interplay indirectly. The optimal weights between the basal and IMRT plans must be further investigated. The proposed method is an alternative to conventional applications in a machine equipped with only FFF beams for treating tumors (other than breast cancer) located at different body parts.

## 5 CONCLUSIONS

This study developed an efficient and practical hybrid method for breast radiotherapy using Halcyon. The results reveal that for patients with early‐stage breast cancer, the proposed method maintained the irradiation time within 90 s—up to 6.5× faster—while providing a comparable PTV coverage, HI, and OAR dose statistics to the DFB‐FiF method. In addition, the workflow in the proposed method can be used to efficiently create PTVs and obviates the requirement of additional CBCT imaging. The proposed method can be an alternative to the DFB‐based method for conventional treatment techniques in the Halcyon machine.

## CONFLICT OF INTEREST

There is no duality/conflict of interest that I should disclose.

## AUTHOR CONTRIBUTION

Yi‐Chun Tsai: contributions to the conception and data acquisition.

Chang‐Shiun Lin: contributions to the conception, interpretation, and draft the work.

Chia‐Chun Wang: contributions to the conception.

Chun‐Wei Wang and Hsiang‐Kung Liang: contributions to the design of the work.

Shu‐Fan Wang and Chia‐Jung Wu: contributions to the plan generation and data acquisition.

## References

[1] Parkin DM , Bray F , Ferlay J , Pisani P . Global cancer statistics, 2002. CA Cancer J Clin. 2005;55:74–108. 15761078 10.3322/canjclin.55.2.74

[2] Fitzmaurice C , Akinyemiju TF , Al Lami FH , et al. Global, regional, and national cancer incidence, mortality, years of life lost, years lived with disability, and disability‐adjusted life‐years for 29 cancer groups, 1990 to 2016: a systematic analysis for the global burden of disease study. JAMA Oncol. 2018;4:1553–1568. 29860482 10.1001/jamaoncol.2018.2706 PMC6248091

[3] Huang C‐S , Lin C‐H , Lu Y‐S , Shen C‐Y . Unique features of breast cancer in Asian women—breast cancer in Taiwan as an example. J Steroid Biochem Mol. 2010;118:300–303. 10.1016/j.jsbmb.2009.12.017 20045728

[4] Veronesi U , Marubini E , Mariani L , et al. Radiotherapy after breast‐conserving surgery in small breast carcinoma: long‐term results of a randomized trial. Ann Oncol. 2001;12:997–1003. 11521809 10.1023/a:1011136326943

[5] Vinh‐Hung V , Verschraegen C . Breast‐conserving surgery with or without radiotherapy: pooled‐analysis for risks of ipsilateral breast tumor recurrence and mortality. J Natl Cancer Inst. 2004;96:115–121. 14734701 10.1093/jnci/djh013

[6] Poggi MM , Danforth DN , Sciuto LC , et al. Eighteen‐year results in the treatment of early breast carcinoma with mastectomy versus breast conservation therapy. Cancer. 2003;98:697–702. 12910512 10.1002/cncr.11580

[7] Tanaka H , Hayashi S , Hoshi H . Determination of the optimal method for the field‐in‐field technique in breast tangential radiotherapy. J Radiat Res. 2014;55:769–773. 24536020 10.1093/jrr/rrt233 PMC4099991

[8] Bortfeld T . IMRT: a review and preview. Phys Med Bio. 2006;51:R363–R379. 16790913 10.1088/0031-9155/51/13/R21

[9] Mihai AM , Sixel KE , Ruschin M , et al. Comparison between forward and inverse planning for breast IMRT. Int J Radiat Oncol Biol Phys. 2003;57:S360.

[10] Selvaraj RN , Beriwal S , Pourarian RJ , et al. Clinical implementation of tangential field intensity modulated radiation therapy (IMRT) using sliding window technique and dosimetric comparison with 3D conformal therapy (3DCRT) in breast cancer. Med Dosim. 2007;32:299–304. 17980832 10.1016/j.meddos.2007.03.001

[11] George R , Keall PJ , Kini VR , et al. Quantifying the effect of intrafraction motion during breast IMRT planning and dose delivery. Med Phys. 2003;30:552–562. 12722807 10.1118/1.1543151

[12] Seco J , Sharp GC , Turcotte J , Gierga D , Bortfeld T , Paganetti H . Effects of organ motion on IMRT treatments with segments of few monitor units. Med Phys. 2007;34:923–934. 17441238 10.1118/1.2436972 PMC2034283

[13] Mayo CS , Urie MM , Fitzgerald TJ . Hybrid IMRT plans—concurrently treating conventional and IMRT beams for improved breast irradiation and reduced planning time. Int J Radiat Oncol Biol. 2005;61:922–932. 10.1016/j.ijrobp.2004.10.033 15708276

[14] Li T , Scheuermann R , Lin A , et al. Impact of multi‐leaf collimator parameters on head and neck plan quality and delivery: a comparison between Halcyon™ and Truebeam® treatment delivery systems. Cureus. 2018;10:e3648. 30723647 10.7759/cureus.3648 PMC6351111

[15] Li T , Irmen P , Liu H , et al. Dosimetric performance and planning/delivery efficiency of a dual‐layer stacked and staggered MLC on treating multiple small targets: a planning study based on single‐isocenter multi‐target stereotactic radiosurgery (SRS) to brain metastases. Front Oncol. 2019;9:7. 10.3389/fonc.2019.00007 PMC6349708 30723702

[16] Lim TY , Dragojević I , Hoffman D , Flores‐Martinez E , Kim G‐Y . Characterization of the Halcyon™ multileaf collimator system. J Appl Clin Med Phys. 2019;20:106–114. 30889312 10.1002/acm2.12568 PMC6448159

[17] Kretschmer M , Sabatino M , Blechschmidt A , Heyden S , Grünberg B , Würschmidt F . The impact of flattening‐filter‐free beam technology on 3D conformal RT. Radiat Oncol. 2013;8:133. 23725479 10.1186/1748-717X-8-133 PMC3695843

[18] Constantin M , Kauppinen J , Kuusela E , Thompson S . A novel dynamic beam flattening technique for 6FFF beams on Halcyon linacs. Med Phys. 2018;45:E497–E498.

[19] Morris R , Laugeman E , Hilliard J , et al. Field‐in‐field breast planning for a jawless, double‐stack MLC LINAC using flattening‐filter‐free beams. J Appl Clin Med Phys. 2019;20:14–26. 10.1002/acm2.12722 PMC6839381 31617671

[20] Cai B , Laugeman E , Mazur TR , et al. Characterization of a prototype rapid kilovoltage x‐ray image guidance system designed for a ring shape radiation therapy unit. Med Phys. 2019;46:1355–1370. 30675902 10.1002/mp.13396 PMC8188470

[21] Lu J‐Y , Zhang J‐Y , Li M , et al. A simple optimization approach for improving target dose homogeneity in intensity‐modulated radiotherapy for sinonasal cancer. Sci Rep. 2015;5:15361. 26497620 10.1038/srep15361 PMC4620500

[22] Ju E , Heo EJ , Park CG , et al. Dosimetric comparison of VitalBeam® and HalcyonTM 2.0 for hypofractionated VMAT with simultaneous integrated boost treatment of early‐stage left‐sided breast cancer. J Appl Clin Med Phys. 2021;22:232–238. 10.1002/acm2.13428 PMC8504599 34554605

[23] Al‐Rahbi ZS , Al Mandhari Z , Ravichandran R , et al. Dosimetric comparison of intensity modulated radiotherapy isocentric field plans and field in field (FIF) forward plans in the treatment of breast cancer. J Med Phys. 2013;38:22. 23531607 10.4103/0971-6203.106601 PMC3607341

